# Whole blood lead concentrations in children undergoing autism assessment in a community paediatric clinic: a retrospective cross-sectional study

**DOI:** 10.1136/bmjpo-2024-003268

**Published:** 2025-07-07

**Authors:** Kirsty Brown, Caroline M Taylor, Richard Lee-Kelland

**Affiliations:** 1 Sirona Care and Health CIC, Bristol, UK; 2 Centre for Academic Child Health, Bristol Medical School, University of Bristol, Bristol, UK

**Keywords:** Child Health, Noncommunicable Diseases

## Abstract

**Objective:**

To investigate blood lead concentrations (BLCs) in children presenting with autistic spectrum disorder (ASD) and associations with clinical presentation (pica, motor delay, language delay and anaemia), age and social deprivation.

**Setting:**

Community-based autism assessment clinics, north Bristol, UK (single-centre, retrospective cross-sectional study).

**Patients:**

Children with autism who had BLC measured as part of an autism assessment during a 4-year period from November 2019 to November 2023.

**Main outcome measures:**

Data were collected from electronic case notes for children who underwent an assessment for ASD during this period, including diagnoses and investigations.

**Results:**

13/102 (13%) children with a diagnosis of autism had BLC ≥0.24 µmol/L, which is above the UK Health Security Agency threshold to trigger further investigation and identification of sources of exposure. Elevated BLC was not associated with the presence of pica or other clinical features including developmental delay.

**Conclusion:**

Pica and developmental delay were not useful indicators of children with elevated BLC. Their absence could lead to cases of elevated BLC being missed in children with autism. This lends weight to an argument that lead should be screened for routinely in the preschool autism population alongside other common causes of behavioural difficulties and developmental delay such as anaemia.

**WHAT IS ALREADY KNOWN ON THIS TOPIC**

- Children with behavioural and/or developmental problems including autism spectrum disorder (ASD) are more likely to have a higher blood lead concentrations (BLCs) than other children. Our aim was to provide an insight into the BLC of children presenting for ASD assessment with a view to informing guidance on screening protocols.

**WHAT THIS STUDY ADDS**

- We show that pica and developmental delay were not useful indicators of children with elevated BLC, and their absence could lead to cases of elevated BLC being missed in children with ASD.

**HOW THIS STUDY MIGHT AFFECT RESEARCH, PRACTICE OR POLICY**

- This study demonstrates that clear and consistent national guidance is required on when clinicians should order BLC measurements in children with ASD.

## Introduction

 Lead is a toxic metal that is found in a variety of everyday substances such as soil, dust, paint and water supplies. Chronic lead exposure presents clinically with a range of features, such as anorexia, headache, abdominal pain, constipation, weight loss, failure to thrive and anaemia (due to lead-induced inhibition of haem synthesis), as well as neurodevelopmental deficits including a reduction in IQ.^1 2^

Lead can be absorbed by ingestion and inhalation of lead-containing particles. Lead exposure is likely to be higher in young children due to hand to mouth behaviour and greater contact with dust and soil if crawling. Children also absorb a greater proportion of lead ingested through the gastrointestinal tract than do adults. Children may be exposed to lead from soil, diet and water (particularly in houses with lead pipes), toys that do not comply with safety regulations, some natural remedies, and renovation work resulting in inhalation of fumes or dust containing lead particles from paint, dust and other sources. High lead exposure may also be associated with pica, which is defined as the eating of non-nutritive non-food substances for at least 1 month, and not in accordance with the child’s developmental stage (age >2 years).^3^ Ingested items can be high in lead, including coal, clay, soil and paint. A recent surveillance report from the UK Health Security Agency found that children with an elevated blood lead concentration (BLC) in England were most commonly exposed to lead in domestic settings (with exposure to paint (43%) and soil (29%)) and usually lived in the most deprived areas of England, generally in rented accommodation.^4^

It has been suggested that children with behavioural and/or developmental problems are more likely to have a higher BLC than other children and therefore warrant routine screening and extended monitoring for lead exposure.^5^ Specifically, several studies have investigated the associations between autism spectrum disorder (ASD) and lead exposure, finding higher concentrations of lead in children with autism but no clear evidence to support causation.^6^,^8^ Even at low exposure levels, lead may be associated with behavioural difficulties, including antisocial behaviour and hyperactivity.^9^ Pica behaviour is a common eating disorder in children with ASD^10^and is characterised by repeated ingestion of non-food items that do not contain significant nutritional value.^11^ Some of these may be contaminated with lead (eg, paint flakes in older houses, dust, soil, coal). In addition, restriction of dietary variety in children with ASD may result in low intakes of calcium and iron, which in turn predisposes children to higher lead absorption because of reduced competition for intestinal binding sites.^12^

Blood tests are not easy to complete in children with autism, and this has led some clinicians to restrict blood lead testing to those children with symptoms of pica or anaemia. The presence of pica could be caused by autism and result in elevated BLC. Alternatively, elevated BLC caused by pica could be causative in the development of ASD. In either case, detection of elevated BLC is essential for treatment and prevention of further exposure to lead.

The UK Health Security Agency currently recommends that clinicians should order a BLC test if a child is showing any pica behaviour and should have a low threshold for screening for lead exposure in children with learning disabilities or behavioural disorders.^13^ They also advise that clinicians should ask parents about risk factors and potential sources of exposure to lead. Specifically, they advise measurement of BLC in children with ASD for those with pica, those who live in housing that contains older building materials or paint that may contain lead, and those who are demonstrating changes in behaviour, delay in development or new gastrointestinal symptoms.

The aim of this study was to provide an insight into the BLC of children presenting for ASD assessment with a view to informing guidance on screening protocols. We aimed to document BLC in children who had blood taken as part of an ASD assessment and specifically to identify the proportion of children who were found to have elevated BLC. Secondary objectives were to identify the proportion that had associated pica, concurrent anaemia and/or speech or motor delay. This study took place in an outpatient community paediatrics setting in north Bristol, UK, where children were referred for an ASD assessment. Data were extracted from electronic records, including confirmed diagnoses of ASD and attention deficit hyperactivity disorder, motor delay, speech delay, pica behaviour, BLC and haemoglobin concentrations, and demographic data including age, sex and postcode.

## Methods

The study was a cross-sectional retrospective review of the electronic records of children (age <18 years) being assessed for ASD within community paediatric clinics in the north Bristol area (UK). Assessments for ASD in this setting are completed initially in a community paediatric outpatient clinic, which is then followed by a structured assessment (ie, Autism Diagnostic Observation Schedule), usually with a speech and language therapist or other trained practitioner, or via a school observation.

We included 147 children who were assessed for ASD over a 4-year period from November 2019 to November 2023 within a single centre in north Bristol and who had blood sampled.

Of these, 102 (69%) had a BLC analysed by inductively coupled plasma mass spectrometry at a local hospital. All 102 children who had a BLC measurement were given a confirmed diagnosis of ASD. An elevated BLC was defined as ≥0.24 µmol/L (cut-off recommended by the UK Health Security Agency for children).^13^

Characteristics extracted from the records were diagnosis, sex, age, postcode, haemoglobin concentration as a marker of anaemia (as defined by the WHO thresholds),^14^ presentation of motor developmental delay and/or speech delay (defined as being identified as an area of developmental delay by the doctor completing the developmental history on the child’s clinic letter) and pica symptoms (defined as the presence of pica symptoms being recorded on the medical record or no pica symptoms being recorded on the medical record). A social deprivation index score was assigned to each child using their home postcode against the Office for National Statistics census data.

Data were analysed with IBM SPSS version for Windows v. 29. Participants were excluded from further analysis if they did not have a BLC measurement. The remaining participants were stratified by BLC <0.24 and ≥0.24 µmol/L. Differences between proportions for characteristics in the two groups were tested with χ^2^ or Fisher’s exact test, or Mann-Whitney U test for continuous variables.

### Public and patient involvement

No patients were involved directly.

## Results

**Figure 1:**
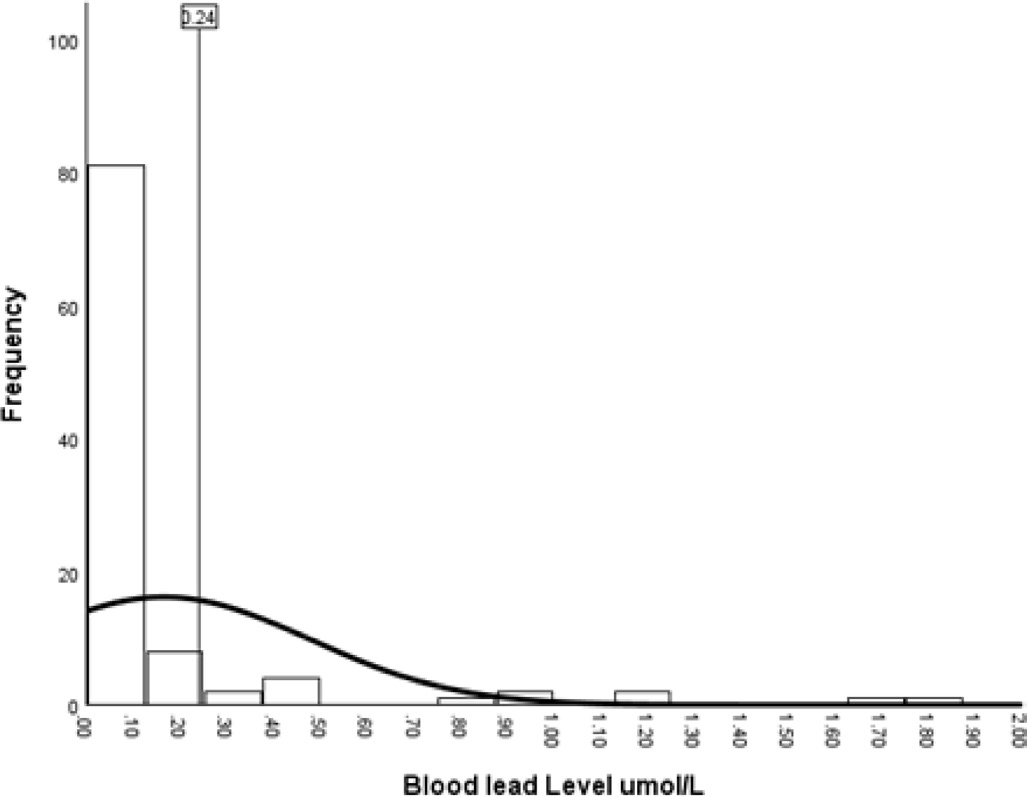
Distribution of blood lead concentrations for the cohort. The dashed vertical line indicates the cut-off for elevated blood lead concentration (≥0.24 µmol/L).

The median BLC was 0.06 (IQR 0.07–0.11) µmol/L (figure 1). In total, 13/102 (12.7%) of the cohort had BLC ≥0.24 µmol/L.

There was no difference in the recorded prevalence of pica in children with BLC <0.24 µmol/L compared with those with BLC ≥0.24 µmol/L (11/89 (12.3%) vs 3/13 (23.1%); p=0.253). Similarly, there was no difference between the proportions of children with motor delay (21/89 (23.6%) vs 4/9 (30.7%); p=0.730) or speech delay (80/89 (89.9%) vs 11/13 (84.6%); p=0.630) (table 1). Anaemia was present in 14/99 (14.1%) of the cohort, but there was no difference in the proportions in the two groups (13/89 (14.6%) vs 1/10 (10.0%); p=1.000) (table 1).

**Table 1:** Characteristics of cohort and characteristics by blood lead concentration (BLC)

Characteristic	Cohort	BLC (μmol/L)		
		<0.24	≥0.24	P value
Sex				
Male (n, %)	72 (71%)	61 (85%)	11 (85%)	
Female (n, %)	30 (29%)	28 (15%)	2 (15%)	0.340
Age (median)	4 years 6 months	4 years 2 months	5 years 6 months	0.080
Social deprivation index decile	2	3	2	0.420
Developmental delay				
Motor delay	25/102 (24.5%)	21/89 (23.6%)	4/9 (30.7%)	0.730
Speech delay	91/102 (89%)	80/89 (89.9%)	11/13 (84.6%)	0.630
Pica (yes)	14/88 (13.7%)	11/89 (12.3%)	3/13 (23.1%)	0.253
Anaemia (yes)	14/99 (14.1%)	13/89 (14.6%)	1/10 (10.0%)	1.000

**Figure 2:**
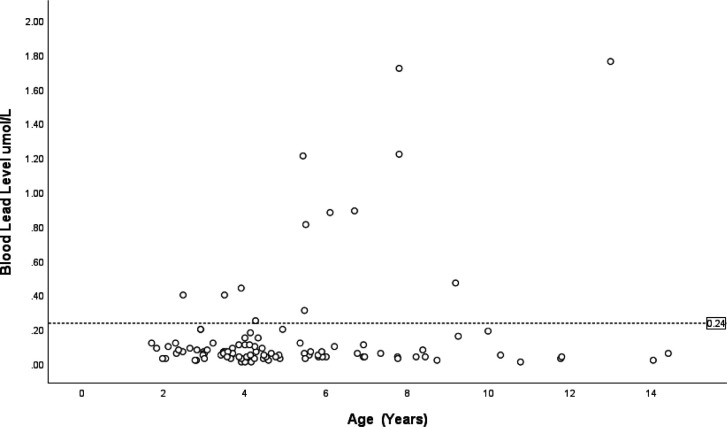
Scatterplot showing blood lead concentration by age (years). The dashed vertical line indicates the cut-off for elevated blood lead concentration (≥0.24 µmol/L).

There was no difference in the age of children with and without high BLC (median 4.19 vs 5.49 years, p=0.080) (figure 2; table 1) or social deprivation index (median 3 vs 2, p=0.420) (table 1).

## Discussion

Overall, 13% of children with a diagnosis of ASD in our sample had a BLC ≥0.24 µmol/L, the concentration at which there should be further investigation due to the possibility of adverse effects on childhood development.^13^ Pica, speech or motor delay, anaemia and socioeconomic status were not associated with elevated BLC. This suggests that merely the absence of these features cannot exclude the possibility of elevated BLC and that this could lead to cases of elevated BLC being missed in children with ASD.

It is estimated that about one in three children globally (up to 800 million) have BLC ≥0.24 µmol/L,^15^ the concentration at which clinical and public health action is advised in many countries, including in the UK for children since 2021.^13^ Children are especially vulnerable to the adverse effects of lead due to the rapid development of their neurological system and lead can have irreversible effects on behaviour and educational attainment.^9^ Hospital activity analysis suggests that about 200 cases of lead poisoning are diagnosed each year in England and Wales.^16^ 340 cases of lead poisoning (≥0.24 µmol/L) were reported in children in England in 2014–2022, predominantly in those aged 1–4 years (54%).^4^ A higher proportion (31%) were living in deprived areas, with pica in 76% of the cohort and learning difficulties in 60%, but there were no data on the prevalence of ASD.^4^

There are few reports of BLC in children with ASD other than single case reports. A meta-analysis including exposure data from studies using hair, blood, urine and teeth indicated that concentrations were elevated in children with ASD compared with controls.^17^ In children who were referred to a US screening clinic because their BLCs were elevated, those who had ASD had a BLC greater than those without ASD (0.88±0.49 vs 0.50±0.31 µmol/L), respectively).^8^

The prevalence of pica in a population sample in preschool children in the UK has been estimated at about 3%.^18^ Our study showed a higher prevalence of pica of 13.7% in this cohort with ASD. A strong association between pica and ASD diagnosis at preschool age (36–115 months) was found in a population-based cohort.^18^ Pica has been found to be associated with undereating, overeating and food fussiness,^18^ and with increased sensory sensitivity traits,^19^ which are traits that are also common in ASD.

The median BLC found in this study (0.06 µmol/L) is greater than the median value reported from the National Health and Nutrition Study in the USA for a population-based sample of children aged 1–5 years in 2018 of 0.03 µmol/L,^20^ but similar to that found in France in children aged 6 months to 6 years in a population sample in 2008/2009 (geometric mean 1.49 µg/dL (0.07 µmol/L)).^21^

Population-level screening is currently not recommended in the UK,^22^ in contrast with routine testing and population sampling in other countries including the USA,^23^ and so the current prevalence of elevated BLC in the overall population of children in the UK is unknown. Data from the UK Avon Longitudinal Study of Parents and Children, an observational birth cohort, in the early 1990s showed that 27% of children age 30 months (total n=582) resident in the Bristol area had BLC ≥0.24 µmol/L,^9^ compared with only 13% in our sample reported here from 2019 to 2023. The most recent population level data in England are from the Health for England Survey 1995: this did not include children, but 752/2563 (29%) of men and 353/2763 (13%) of women had BLC ≥0.24 µmol/L.^24 25^

It is likely that BLCs in the UK have declined since the 1990s following the banning of lead in paint and petrol, as has been found in other countries such as the USA^26^ and other European countries,^27^ but significant sources of lead remain in the child’s environment, including old paint, lead pipes and historic dust in older houses, soil, passive smoking, and other natural and anthropogenic sources.

The decision to order a BLC in this study was taken by the individual clinician assessing the case. UK national guidance does not stipulate a particular blood test as part of the investigation of ASD.^28^ The local policy (Sirona Care & Health) is that BLC testing can be ordered as part of investigations into developmental delay for children (<18 years old). However, BLC is not always included in this investigation as there is variation between clinicians as to whether a test will be ordered as part of developmental screening, with some clinicians only requesting BLC when there is evidence of pica and/or anaemia.

The strengths of our study are in adding to a relatively small body of literature on BLC in children with ASD in a well-defined clinical population. This is an area of significant concern for families and clinicians and our study provides a large sample with concurrent information on their presentation including clinical information on developmental delay and pica. The results may help produce clinical guidance and inform practice in this area.

There are some limitations to this study. First, our results may not be generalisable to the rest of the UK or to the wider overall population of children with ASD in the UK. Our data were collected in a single centre, retrospective design study that may not be representative of children across the UK. In addition, Bristol has a history of lead mining and related lead-working industries that may affect local lead exposure levels.^29^

Second, our cohort is likely to represent a subset of ASD that is younger with a higher prevalence of developmental delay than the paediatric ASD population as a whole, because investigation of developmental delay was generally the trigger for blood testing. Not all children who presented for an ASD assessment had blood samples taken and within our sample: BLCs were measured as part of the blood tests completed in only 69% of our cohort. This could have introduced further bias into our sample. It was not possible for us to retrospectively make an accurate judgement of the number of children who may have been assessed for autism over the period of the study or, if identified, it would not be possible to identify if they have raised BLC at the time of their assessment. There was no standardised question for pica in the history taking, or obligation to record the presence or absence of the behaviour in the clinical notes.

Third, ideally we would like to have formalised the identification of developmental delay by using a structured assessment tool such as the Schedule of Growing Skills or Bayley Scales. However, these are not standard tools for clinical assessments in our clinic given the pressures on service and our study instead relies on a clinician taking a developmental history and observation in clinic to make an assessment on the degree of developmental delay.

Finally, the study design does not permit conclusions of causation around whether lead exposure causes ASD or whether ASD increases the likelihood of lead exposure (eg, by increasing likelihood of eating flakes of leaded paint) or a combination of the two. We also lack a comparison with a control group of children without ASD. Retrospective power calculations for cross-sectional study designs indicated that our study was underpowered to detect differences in the clinical presentation variables.^30^ However, it would be unfeasible to wait for a sufficient number of children with a diagnosis of ASD at the clinic (eg, >2000 for speech delay). Our results should therefore be interpreted with caution. However, although the study is underpowered, it still has clinical utility, and it does appear from our results that reliance of the absence of pica and/or developmental delay is not sufficient to exclude high BLC and doing so may lead to cases being missed—to the potential detriment of children.

## Conclusion

In this clinic sample, raised BLCs were seen commonly when requested, and 13% of this cohort had a level that required further investigation through public health services. The absence of pica or developmental delay did not preclude measurement of BLC, and it is possible that cases might be missed if testing was not completed due to the absence of these features (in our sample, 12% with no pica recorded had a high BLC compared with 23% with pica recorded). This lends weight to an argument that testing of BLC should be included when taking bloods as part of an autism assessment rather than restricting blood testing to only children with pica. Clear and consistent national guidance is required on when clinicians should order BLC measurements in children with ASD.

## Data Availability

No data are available.
