# Supplementary material for: Whole blood lead concentrations in children undergoing autism assessment in a community paediatric clinic: a retrospective cross-sectional study
Source: BMJ Paediatr Open. 2025 Jul 7;9(1):e003268. doi: 10.1136/bmjpo-2024-003268 (PMC12232456; doi:10.1136/bmjpo-2024-003268)
Supplement: Supplementary file 1 [file bmjpo-2024-003268.reviewer_comments.pdf]

PEER REVIEW HISTORY

BMJ Paediatrics Open publishes all reviews undertaken for accepted manuscripts. Reviewers are asked to complete a checklist review form and are provided with free text boxes to elaborate on their assessment. These free text comments are reproduced below.

ARTICLE DETAILS

**TITLE (PROVISIONAL)**	Whole blood lead concentrations in children undergoing autism assessment in a community paediatric clinic: a retrospective cross-sectional study
**AUTHORS**	Brown, Kirsty; Taylor, Caroline; Lee-Kelland, Richard

VERSION 1 - REVIEW

**REVIEWER NAME**	***Hilary Branch***
**REVIEWER AFFILIATION**	Baystate Medical Center Children's Hospital, Pediatrics
**REVIEWER CONFLICT OF INTEREST**	N/A
**AI DISCLOSURE**	No
**DATE REVIEW RETURNED**	02-Feb-2025

**GENERAL COMMENTS**	Glad you are doing a study addressing the importance of screening for lead poisoning in children with autism.<br><br>You should mention in the introduction that even at low levels, lead can cause behavior problems such as ADHD and aggressive behavior .(This potentially could worsen behavior concerns which often are already challenging.)<br><br>You mentioned in the discussion a limitation - that 69% of the children being assessed for lead poisoning had a lead level drawn. Expanding on this that the sample could have been biased would be important.<br><br>Also the limitation that there was no definition of PICA is important. You should comment that the amount of hand to mouth activity was not addressed. Hand to mouth activity with or without PICA has been shown to be a a risk.<br><br>You still can come to your conclusion assuming the standard definition of PICA- eating non food items was asked. This is important if providers are using the presence of PICA in their decision on whether to screen.<br><br>In the conclusion I would stop after the statement about not limiting lead testing in patents with PICA. .. Routine screening for lead exposure in this population should be considered. Further studies should be done to evaluate this.<br>The last sentence is vague and can be interpreted as being discriminatory against children with this disability.

**REVIEWER NAME**	***Calum McPherson***
**REVIEWER AFFILIATION**	NHS Greater Glasgow and Clyde

**REVIEWER CONFLICT OF INTEREST**	N/A
**AI DISCLOSURE**	No
**DATE REVIEW RETURNED**	16-May-2025

**GENERAL COMMENTS**	This study adds to the small body of literature on lead exposure in the context of autism spectrum disorder. I recommend for publication with minor revisions.<br><br>The study addresses a clearly focussed issue: blood lead concentrations in children presenting with autism spectrum disorder and investigates associations with clinical presentation, age and social deprivation.<br><br>This was a cross-sectional study - an appropriate study method to address the study question. The paper is described as a cohort study in the abstract – can the authors change this description to cross-sectional study?<br><br>In terms of participants, authors included “147 children who were assessed for ASD over a 4-year period… and who had blood sampled”. Could the authors clarify whether this represents all children assessed for ASD in this setting during this time period, or whether this was a subset of the total? If a subset of the total, were groups comparable? Of those assessed for who ASD who did not have blood sampled (if any), were the demographics, clinical presentation and social deprivation comparable to those who did blood sampled?<br><br>The setting for data collection was appropriate.<br><br>In terms of measurement and classification bias, some measures were accurately measured and some were not. It is clearly described how some objective measures (age, lead levels, deprivation levels) were obtained. Lead levels were measured using a standard ICP-MS process and Hb concentrations were used to determine anaemia, as defined by WHO thresholds – both can be considered accurate methods of measurement. Social deprivation index scores were assigned using home postcode and ONS census data – this accounts of area-wide deprivation but is a typical proxy for individual deprivation in cross-sectional studies.<br><br>Pica, motor delay and language delay were not assessed or recorded in standard objective formats in the accessed medical records. This, along with consequent subjective interpretation of notes for these classifications may have introduced bias. Authors clearly acknowledge this in the discussion section. Could the authors clarify how the medical records were interpreted for developmental delay and pica? Would a description of inability to do X at Y age be interpreted as delay by the study authors using a standardised developmental assessment tool, or did study authors rely exclusively on a stated clinician opinion of delay in a specific domain?<br><br>Authors did not describe a power or sample size calculation. It is unclear if the study is appropriately powered/the sample size is large enough to justify statistical analysis and answer its objectives. Could the authors include a power or sample size calculation with acknowledgement if the study is underpowered to answer its objectives?

	The results are clearly presented as proportions with percentages in brackets, along with an associated p-number. Described statistical tests were appropriate.<br>There are statements of findings which somewhat misrepresent results. The study states "There was no difference…" on three occasions when describing results. Could the authors clarify that there was no statistically significant difference between groups, as the study may not be powered to draw the conclusion of no actual difference?<br><br>In the discussion, study authors state "pica, speech or motor delay, anaemia and socio-economic status were not associated with elevated BLC." This somewhat misrepresents results as the paper does not report if the study is powered to determine these associations. Study authors report that the study design does not permit conclusions of causation. Could the authors clarify that the design does not permit conclusions of association?<br><br>Study authors have nicely discussed findings in the local, global and historical contexts. The conclusion appropriately summarises the study findings, along with highlighting areas of need including clear and consistent national guidance.

VERSION 1 – AUTHOR RESPONSE

**COMMENT**	**RESPONSE**
• Patient and Public Involvement<br>Authors must include a statement in the methods section of the manuscript under the sub-heading 'Patient and Public Involvement'.<br><br>This should provide a brief response to the following questions:<br><br>How was the development of the research question and outcome measures informed by patients' priorities, experience, and preferences?<br>How did you involve patients in the design of this study?<br>Were patients involved in the recruitment to and conduct of the study?<br>How will the results be disseminated to study participants?<br>For randomised controlled trials, was the burden of the intervention assessed by patients themselves?<br>Patient advisers should also be thanked in the contributorship statement/acknowledgements.<br><br>If there is no patient involved in the study, please state "No patient involved" under the sub-heading 'Patient and public involvement'.	The heading 'Public and patient involvement' has been included in the methods. The text 'No patient involved' has been added.

COMMENT	RESPONSE
• Key message<br>Please include the key messages of your article after your abstract using the following headings. This section should be no more than 3-5 sentences and should be distinct from the abstract; be succinct, specific and accurate.<br><br>-What is already known on this topic – summarise the state of scientific knowledge on thics subject before you did your study and why this study needed to be done<br>-What this study adds – summarise what we now know as a result of this study that we did not know before<br>-How this study might affect research, practice or policy – summarise the implications of this study<br><br>This will be published as a summary box after the abstract in the final published article.	Key messages were already included after the abstract:<br><br>**Key messages**<br><br>• Children with behavioural and/or developmental problems including autism spectrum disorder (ASD) are more likely to have a higher blood lead concentrations (BLC) than other children and may therefore warrant routine screening and extended monitoring for lead exposure<br>• Pica and developmental delay were not useful indicators of children with elevated BLC and their absence could lead to cases of elevated BLC being missed in children with ASD<br>• Clear and consistent national guidance is required on when clinicians should order BLC measurements in children with ASD
Additionally the editorial team would like you to address the following and respond:<br>*1. The IRAS ID does not correspond to this study. I know that some sub-studies can be under an umbrella IRAS ID but the linked IRAS ID seems entirely unrelated.*	The IRAS ID is for the PALE study into childhood anaemia, which included testing of lead levels as a cause of anaemia. An amendment to the IRAS was made to create a comparison database of all children having lead levels completed as part of an autism assessment, which was required to interpret the results of the PALE study. The data from this is assessed in this study, which looks at all lead levels used in that database.
2. One of the authors has a private practice which conducts autism assessments. Co-located private children's services offer blood tests. Theoretically, his organisation could financially benefit from recommendations expanding routine blood tests in the context of autism. I think there is an expectation that private practice in the field of interest is disclosed.	As requested we have disclosed that one of the authors undertakes private practice around autism assessments, reproduced below:<br><br>RL-K undertakes private practice in addition to his NHS work around assessment of autism.
**REVIEWER 1**	

COMMENT	RESPONSE
You should mention in the introduction that even at low levels, lead can cause behavior problems such as ADHD and aggressive behavior .(This potentially could worsen behavior concerns which often are already challenging.)	Thank you for this suggestion. The following sentence has been added to the Introduction:<br><br>Even at low exposure levels, lead may be associated with behavioural difficulties, including antisocial behaviour and hyperactivity[9].
You mentioned in the discussion a limitation - that 69% of the children being assessed for lead poisoning had a lead level drawn. Expanding on this that the sample could have been biased would be important.	A new sentence has been added to indicate that this could have introduced further bias into our sample.
Also the limitation that there was no definition of PICA is important. You should comment that the amount of hand to mouth activity was not addressed. Hand to mouth activity with or without PICA has been shown to be a a risk.<br><br>You still can come to your conclusion assuming the standard definition of PICA- eating non food items was asked. This is important if providers are using the presence of PICA in their decision on whether to screen.	The DSM-5 defines pica as 'an eating disorder that involves eating items that are not typically thought of as food and that do not contain significant nutritional value, such as hair, dirt, and paint chips'. This definition and reference citation have been added in the Introduction.
In the conclusion I would stop after the statement about not limiting lead testing in patents with PICA. .. Routine screening for lead exposure in this population should be considered. Further studies should be done to evaluate this.<br>The last sentence is vague and can be interpreted as being discriminatory against children with this disability.	Thank you for this point. The final sentence has been deleted.
**REVIEWER 2**	
This was a cross-sectional study - an appropriate study method to address the study question. The paper is described as a cohort study in the abstract – can the authors change this description to cross-sectional study?	Thank you. We have changed the study design from cohort study to cross-sectional in the title and abstract.

COMMENT	RESPONSE
In terms of participants, authors included “147 children who were assessed for ASD over a 4-year period… and who had blood sampled”. Could the authors clarify whether this represents all children assessed for ASD in this setting during this time period, or whether this was a subset of the total? If a subset of the total, were groups comparable? Of those assessed for who ASD who did not have blood sampled (if any), were the demographics, clinical presentation and social deprivation comparable to those who did blood sampled?	The 147 children who were sampled does not reflect all children locally who were assessed for autism over this period. This reflects current clinical practice. Blood tests (including lead levels) are sometimes requested as part of an autism assessment but are not a standard investigation for autism, and there is no national guidance as to when they should be performed. They are requested on the discretion of individual clinicians (generally on the basis of symptoms such as developmental delay or pica). This is a weakness of the study, and we reflect on this in the discussion and have inserted further text below to indicate that this is a potential source of bias:<br><br>Second, our cohort is likely to represent a subset of ASD that is younger with a higher prevalence of developmental delay than the paediatric ASD population as a whole, because investigation of developmental delay was generally the trigger for blood testing. Not all children who presented for an ASD assessment had blood samples taken and within our sample, BLC were measured as part of the blood tests completed in only 69% of our cohort. This could have introduced further bias into our sample. It was not possible for us to retrospectively make an accurate judgment of the number of children who may have been assessed for autism over the period of the study or, if identified, it would not be possible to identify if they have raised BLC at the time of their assessment.
Pica, motor delay and language delay were not assessed or recorded in standard objective formats in the accessed medical records. This, along with consequent subjective interpretation of notes for these classifications may have introduced bias. Authors clearly acknowledge this in the discussion section. Could the authors clarify how the medical records were interpreted for developmental delay and pica? Would a description of inability to do X at Y age be interpreted as delay by the study authors using a standardised developmental assessment tool, or	This is a source of bias that we have identified and is reflected on in the discussion section. Due to the limitations of a clinic appointment there is not the time to complete an objective assessment of development (i.e. Bayley or Griffiths scales) and there is no standard form to record the presence or absence of symptoms. Clinicians made their assessment of development delay/pica based on history and examination in clinic which reflects current clinical practice.

COMMENT	RESPONSE
did study authors rely exclusively on a stated clinician opinion of delay in a specific domain?	In our study the medical notes were retrospectively reviewed by a paediatrician to see if the was clinical evidence of pica and/or developmental delay from the notes, including a stated opinion from the assessing doctor.
Authors did not describe a power or sample size calculation. It is unclear if the study is appropriately powered/the sample size is large enough to justify statistical analysis and answer its objectives. Could the authors include a power or sample size calculation with acknowledgement if the study is underpowered to answer its objectives?	Our null hypothesis was that in children with ASD, clinical presentation (pica, motor delay, language delay, anaemia) was not associated with BLC above 0.24.<br><br>Motor delay: for 80% power with 31% of sample with BLC>0.24 having motor delay, the total sample size required would be 2712.<br><br>Speech delay: 4024<br><br>Pica: 718<br><br>Anaemia: 4024<br><br>OpenEpi:Sample Size for X-Sectional,Cohort,and Clinical Trials<br><br>We readily acknowledge that our study is underpowered according to this retrospective power calculation. We have added the following text to the limitations in the Discussion.<br><br>Retrospective power calculations for cross-sectional study designs indicated that our study was underpowered to detect differences in the clinical presentation variables[30]. However, it would be unfeasible to wait for a sufficient number of children with a diagnosis of ASD at the clinic (e.g. >2000 for speech delay). Our results should therefore be interpreted with caution. However, although the study is underpowered it still has clinical utility, it does appear from our results that reliance of the absence of pica and/or developmental delay is not sufficient to exclude high BLC and doing so may lead to

<table>
<tr><th>COMMENT</th><th>RESPONSE</th></tr>
<tr><td></td><td>cases being missed – to the potential detriment of children</td></tr>
<tr><td>The results are clearly presented as proportions with percentages in brackets, along with an associated p-number. Described statistical tests were appropriate.
There are statements of findings which somewhat misrepresent results. The study states “There was no difference…” on three occasions when describing results. Could the authors clarify that there was no statistically significant difference between groups, as the study may not be powered to draw the conclusion of no actual difference?</td><td>The reviewer is likely referring to these portions of text:

• There was no difference in the recorded prevalence of pica in children with BLC <0.24 μmol/L compared with those with BLC ≥0.24 μmol/L (11/89 (12.3%) vs 3/13 (23.1%); p=0.253).
• Similarly, there was no difference between the proportions of children with motor delay (21/89 (23.6%) vs 4/9 (30.7%); p=0.730) or speech delay (80/89 (89.9%) vs 11/13 (84.6%); p=0.630) (Table 1).
• Anaemia was present in 14/99 (14.1%) of the cohort but there was no difference in the proportions in the two groups (13/89 (14.6%) vs 1/10 (10.0%); p=1.000) (Table 1).
• There was no difference in the age of children with and without high BLC (median 4.19 vs 5.49 years, p=0.080 (Figure 2; Table 1) or social deprivation index (median 3 vs 2, p=0.420) (Table 1).

In each instance, the p value has been provided, indicating the strength of evidence against the null hypothesis (one value does not affect the other). In each instance, the p values are high, indicating that there was no difference between the two values in each pair (we prefer not to use the term ‘statistically significantly different’ as it relies on a cut-off at an arbitrary level of p<0.05).

Sifting the evidence—what's wrong with significance tests? - PMC</td></tr>
<tr><td>In the discussion, study authors state “pica, speech or motor delay, anaemia and socio-economic status were not associated with elevated BLC.” This somewhat misrepresents results as the paper does not report if the study is powered to determine these associations.</td><td>Thank you for this comment. We use the term ‘associated with’ deliberately as it does not imply causation and is cautious. It simply implies that we did not identify a relationship between two variables without direction in this study. We note in the final paragraph of the Discussion that the study design does not</td></tr>
</table>

COMMENT	RESPONSE
Study authors report that the study design does not permit conclusions of causation. Could the authors clarify that the design does not permit conclusions of association?	allow a definitive exclusion of causation (and therefore association as you point out).<br><br>Although the study is underpowered (and producing a study powered enough to answer the question would be technically challenging) the study does provide clinically useful information in that it demonstrates that many children had raised lead levels did not have clinical signs that are thought to be indicative of the condition (anaemia, pica and developmental delay) – in fact we saw little difference between these groups and therefore reliance on these symptoms may lead to missed cases of high lead levels.
